# Supplementary material for: Resolving the full spectrum of human genome variation using Linked-Reads
Source: Genome Res. 2019 Apr;29(4):635–45. doi: 10.1101/gr.234443.118 (PMC6442396; doi:10.1101/gr.234443.118)
Supplement: Supplemental Material [file supp_gr.234443.118_Supplemental_longranger-2.2.zip › longranger-2.2.tar.bz2!/lr2.2-public/lib/stan/cmdstan/stan/doc/api/html/structstan_1_1gm_1_1functions__grammar.html]

Stan: stan::gm::functions\_grammar< Iterator > Struct Template Reference


|  |  |
| --- | --- |
|  | Stan  2.5.0  probability, sampling & optimization |


- Main Page
- Related Pages
- Namespaces
- Classes
- Files

- Class List
- Class Index
- Class Hierarchy
- Class Members

All Classes Namespaces Files Functions Variables Typedefs Enumerations Enumerator Friends Macros Pages

- stan
- gm
- functions\_grammar

Public Member Functions |
Public Attributes |
List of all members

stan::gm::functions\_grammar< Iterator > Struct Template Reference

`#include <functions_grammar.hpp>`

Inheritance diagram for stan::gm::functions\_grammar< Iterator >:

|  |  |
| --- | --- |
| Public Member Functions | |
|  | functions\_grammar (variable\_map &var\_map, std::stringstream &error\_msgs) |
|  | |

|  |  |
| --- | --- |
| Public Attributes | |
| variable\_map & | var\_map\_ |
|  | |
| std::set< std::pair  < std::string,   function\_signature\_t > > | functions\_declared\_ |
|  | |
| std::set< std::pair  < std::string,   function\_signature\_t > > | functions\_defined\_ |
|  | |
| std::stringstream & | error\_msgs\_ |
|  | |
| statement\_grammar< Iterator > | statement\_g |
|  | |
| bare\_type\_grammar< Iterator > | bare\_type\_g |
|  | |
| boost::spirit::qi::rule  < Iterator, std::vector  < function\_decl\_def >  ), whitespace\_grammar  < Iterator > > | functions\_r |
|  | |
| boost::spirit::qi::rule  < Iterator,   boost::spirit::qi::locals  < bool, int >  , function\_decl\_def(),   whitespace\_grammar< Iterator > > | function\_r |
|  | |
| boost::spirit::qi::rule  < Iterator, std::vector  < arg\_decl >  ), whitespace\_grammar  < Iterator > > | arg\_decls\_r |
|  | |
| boost::spirit::qi::rule  < Iterator, arg\_decl(),   whitespace\_grammar< Iterator > > | arg\_decl\_r |
|  | |
| boost::spirit::qi::rule  < Iterator, std::string(),   whitespace\_grammar< Iterator > > | identifier\_r |
|  | |

## Detailed Description

### template<typename Iterator> struct stan::gm::functions\_grammar< Iterator >

Definition at line 21 of file functions\_grammar.hpp.

## Constructor & Destructor Documentation

template<typename Iterator >

|  |  |  |  |
| --- | --- | --- | --- |
| stan::gm::functions\_grammar< Iterator >::functions\_grammar | ( | variable\_map & | *var\_map*, |
|  |  | std::stringstream & | *error\_msgs* |
|  | ) |  |  |

Definition at line 290 of file functions\_grammar\_def.hpp.

## Member Data Documentation

template<typename Iterator >

boost::spirit::qi::rule<Iterator, arg\_decl(), whitespace\_grammar<Iterator> > stan::gm::functions\_grammar< Iterator >::arg\_decl\_r

Definition at line 66 of file functions\_grammar.hpp.

template<typename Iterator >

boost::spirit::qi::rule<Iterator, std::vector<arg\_decl>), whitespace\_grammar<Iterator> > stan::gm::functions\_grammar< Iterator >::arg\_decls\_r

Definition at line 61 of file functions\_grammar.hpp.

template<typename Iterator >

bare\_type\_grammar<Iterator> stan::gm::functions\_grammar< Iterator >::bare\_type\_g

Definition at line 40 of file functions\_grammar.hpp.

template<typename Iterator >

std::stringstream& stan::gm::functions\_grammar< Iterator >::error\_msgs\_

Definition at line 36 of file functions\_grammar.hpp.

template<typename Iterator >

boost::spirit::qi::rule<Iterator, boost::spirit::qi::locals<bool,int>, function\_decl\_def(), whitespace\_grammar<Iterator> > stan::gm::functions\_grammar< Iterator >::function\_r

Definition at line 56 of file functions\_grammar.hpp.

template<typename Iterator >

std::set<std::pair<std::string, function\_signature\_t> > stan::gm::functions\_grammar< Iterator >::functions\_declared\_

Definition at line 32 of file functions\_grammar.hpp.

template<typename Iterator >

std::set<std::pair<std::string, function\_signature\_t> > stan::gm::functions\_grammar< Iterator >::functions\_defined\_

Definition at line 34 of file functions\_grammar.hpp.

template<typename Iterator >

boost::spirit::qi::rule<Iterator, std::vector<function\_decl\_def>), whitespace\_grammar<Iterator> > stan::gm::functions\_grammar< Iterator >::functions\_r

Definition at line 50 of file functions\_grammar.hpp.

template<typename Iterator >

boost::spirit::qi::rule<Iterator, std::string(), whitespace\_grammar<Iterator> > stan::gm::functions\_grammar< Iterator >::identifier\_r

Definition at line 71 of file functions\_grammar.hpp.

template<typename Iterator >

statement\_grammar<Iterator> stan::gm::functions\_grammar< Iterator >::statement\_g

Definition at line 39 of file functions\_grammar.hpp.

template<typename Iterator >

variable\_map& stan::gm::functions\_grammar< Iterator >::var\_map\_

Definition at line 27 of file functions\_grammar.hpp.

---

The documentation for this struct was generated from the following files:

- src/stan/gm/grammars/functions\_grammar.hpp
- src/stan/gm/grammars/functions\_grammar\_def.hpp


---

[ Stan Home Page ]

*© 2011–2014,
Stan Development Team.*
